# Supplementary material for: The causal role of circulating inflammatory markers in osteoporosis: a bidirectional Mendelian randomized study
Source: Front Immunol. 2024 Jul 18;15:1412298. doi: 10.3389/fimmu.2024.1412298 (PMC11291241; doi:10.3389/fimmu.2024.1412298)
Supplement: Supplementary file 1 [file DataSheet_1.zip › supplementary material/CIM-OSTEOPOROSIS /GCST90274792.csv_forest.pdf]

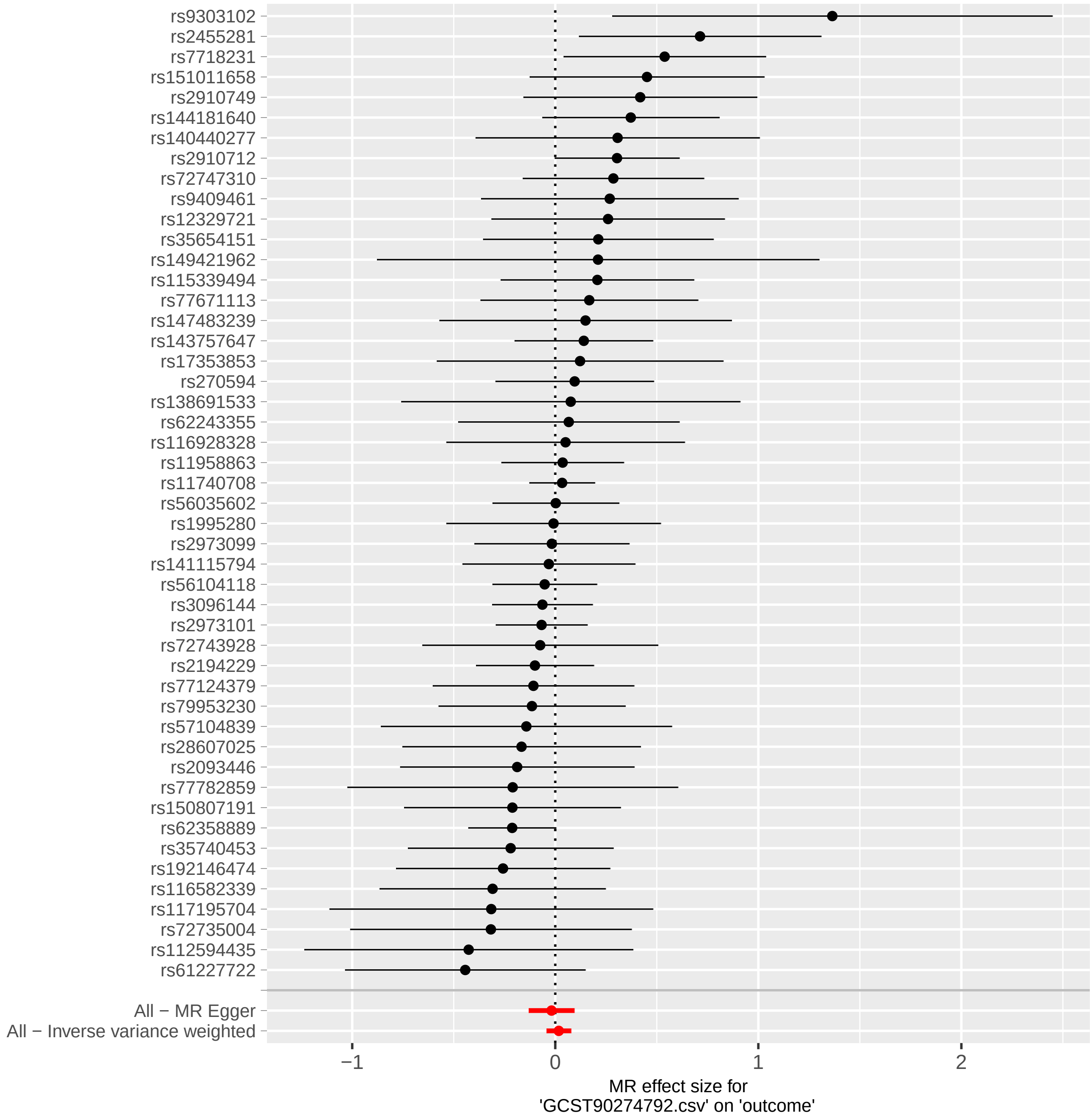

rs9303102
rs2455281
rs7718231
rs151011658
rs2910749
rs144181640
rs140440277
rs2910712
rs72747310
rs9409461
rs12329721
rs35654151
rs149421962
rs115339494
rs77671113
rs147483239
rs143757647
rs17353853
rs270594
rs138691533
rs62243355
rs116928328
rs11958863
rs11740708
rs56035602
rs1995280
rs2973099
rs141115794
rs56104118
rs3096144
rs2973101
rs72743928
rs2194229
rs77124379
rs79953230
rs57104839
rs28607025
rs2093446
rs77782859
rs150807191
rs62358889
rs35740453
rs192146474
rs116582339
rs117195704
rs72735004
rs112594435
rs61227722
All − MR Egger
All − Inverse variance weighted
−1
0
1
2
MR effect size for
'GCST90274792.csv' on 'outcome'
